# Prevalence and Risk Profile of Depression Among Adolescents in Rural and Indigenous Communities of Mexico: A Cross-Sectional Study

**DOI:** 10.1192/j.eurpsy.2025.602

**Published:** 2025-08-26

**Authors:** A. Vega, L. Díaz-Castro, G. B. Ramírez-Rodríguez, K. Hoffman

**Affiliations:** 1 Direction of Epidemiological and Psychosocial Research, National Institute of Psychiatry Ramón de la Fuente Muñiz, Mexico City; 2 Neurogenesis Laboratory, National Institute of Psychiatry Ramón de la Fuente Muñiz, Ciudad de México; 3 Neurogenesis Laboratory, National Institute of Psychiatry Ramón de la Fuente Muñiz, Mexico City; 4 Research Center in Animal Reproduction, Autonomous University of Tlaxcala, Tlaxcala, Mexico

## Abstract

**Introduction:**

Depressive disorders are the most prevalent chronic mental disorders globally, affecting approximately 322 million people, or 4.4% of the world’s population, with a significant portion residing in the Americas, including Mexico. Adolescence represents a critical period for the onset of depression, where preventive interventions should focus on enhancing cognitive abilities, which are malleable and can mitigate the impact of early negative experiences, particularly in marginalized areas and indigenous communities.

**Objectives:**

To identify the prevalence of depressive symptoms and characteristics associated with the presence or absence of depressive symptoms in a population of adolescents and young adults residing in rural and indigenous communities in San Luis Potosi state, Mexico.

**Methods:**

A cross-sectional study was conducted. Depressive symptoms were assessed using the Patient Health Questionnaire-9 (PHQ-9), while anxiety symptoms were measured using the Generalized Anxiety Disorder-7 (GAD-7) scale. Descriptive statistics, a comparative analysis and a principal components analysis were performed with the sociodemographic data and the evaluations of each item of the PHQ9 and GAD7.

**Results:**

1,057 participants aged between 15 and 25 years (16.63 ± 1.53 years) were included in the study. The sample comprised 60.51% females 39.48% males, and 7 participants reported speaking an indigenous language. 28.67% of participants had responses compatible with anxiety, while 34.98% had depression, of which 46.1% qualified as having major depressive disorder. Regarding GAD7, participants with higher severity scores presented a higher average response on item 3 about feeling excessively worried about different things, while those with depression did not respond predominantly to questions regarding mood, but rather to item 3 referring to having difficulty falling or staying asleep and item 4 about feeling tired or having low energy. 4.67% of participants reported suicidal ideation almost every day. When the GAD7 and PHQ9 items were subjected to a principal component analysis, it was observed that PC1=51.67%. The factors self-reported as most closely linked to depressive and anxious symptoms included the age of the caregiver, sex and age of the participant, as well as whether they spoke an indigenous language.

**Conclusions:**

Difficulty falling and staying asleep, as well as perceived lack of energy or fatigue, are the main ways in which this population recognizes signs of depression, rather than feelings of sadness or anhedonia. Given the high prevalence of depressive symptoms and the identified risk profiles, there is an urgent need for targeted mental health services and interventions in these vulnerable populations.

**Disclosure of Interest:**

None Declared

